# Prevalence and Risk Factors of Osteoporosis in Women Referring to the Bone Densitometry Academic Center in Urmia, Iran

**DOI:** 10.5539/gjhs.v8n7p135

**Published:** 2015-11-18

**Authors:** Marzieh Saei Ghare Naz, Giti Ozgoli, Mir Amir Aghdashi, Fatemeh Salmani

**Affiliations:** 1 Department of Midwifery, School of Nursing and Midwifery, Urmia University of Medical Sciences, Urmia, Iran; 2 Department of Midwifery and Reproductive Health, School of Nursing and Midwifery, Shahid Beheshti University of Medical Sciences, Tehran, Iran; 3 Department of Rheumatology, Faculty of Medicine, Urmia University of Medical Sciences, Urmia, Iran; 4 Departments of Biostatistics, Faculty of Biostatistics, Shahid Beheshti University of Medical Sciences, Tehran, Iran

**Keywords:** women, osteoporosis, bone mineral density, risk factor, Iran

## Abstract

**Background:**

Osteoporosis is one of the fastest growing health problems around the world. Several factors can affect this silent disease. The current study aimed to determine the prevalence and risk factors of osteoporosis in women in Urmia, a city in northwestern Iran.

**Methods:**

This cross-sectional study was performed on 360 non-pregnant women over the age of 15 who referred for bone density testing to the Urmia Imam Khomeini Academic Hospital. Data were collected by questionnaire, and bone mineral density of the femoral neck and lumbar spines L1- L4 was evaluated by dual X-ray absorptiometry.

**Results:**

The total prevalence of osteoporosis in this study was 42.2%; prevalence of osteoporosis among women 45 years old or less was 14.3% and over the age of 45 years was 50.7%. The factors such as level of education, history of bone fracture, disease history (rheumatoid arthritis, diabetes, high blood pressure), gravidity and parity values, duration of lactation (p<0.001), nutrition dimension of lifestyle (p=0.03), and green tea consumption (p=002) showed a statistically significant association with the bone mineral density. According to the regression model, age (OR=1.081), history of bone fracture (OR=2.75), and gravidity (OR=1.14) were identified as significant risk factors for osteoporosis, while the body mass index (OR=0.94) was identified as a protector against osteoporosis.

**Conclusion:**

The prevalence of osteoporosis in this study was high, and findings showed that the advancement of age, lifestyle, and reproductive factors (especially gravidity and duration of lactation) were determining factors for osteoporosis. Appropriate educational programs and interventions could help to increase the women’s peak bone mass therefore reducing their risk of developing osteoporosis.

## 1. Introduction

Osteoporosis is a common health problem currently threatening the health of millions of women (Stetzer, 2011). Bone fracture is a first symptom of this silent disease (Willson et al., 2015). Osteoporosis is defined as a disorder with bone mineral density [BMD] 2.5 or more standard deviations less than the mean BMD in healthy young adults [T-score −2.5 or less] (Schousboe & Ensrud, 2015). The peak bone mass of women is lower than that of men; therefore as age advances, women are more likely to be at risk for osteoporosis (Oommen & AlZahrani, 2014). It is estimated that every 3 seconds one osteoporotic fracture occurs somewhere in the world (Stovall, 2013). The universal burden of the low BMD almost doubled (0.12% vs. 0.21%) over the 20–year period from 1990 to 2010, and low BMD caused nearly one-third of the all fall-related deaths around the world (Sanchez-Riera et al., 2014).

Among Iranian women, the prevalence rates of osteoporosis in lumbar vertebrae and the femur are 41.7% and 3.6%, respectively (Maalouf et al., 2007). The Iranian Multi-centers Osteoporosis Study (IMOS) reported that more than two-thirds of the women and half of the men over the age of 50 have a low bone mineral density (Rahnavard et al., 2009). Additionally, according to a meta-analytic study conducted in Iran, low bone mineral density among Iranians older than 30 years is a growing health problem (Irani et al., 2013). It has been estimated that in 2001, osteoporosis led to the loss of 17,270 years of life for Iranian women (Abolhassani et al., 2004). With the increased number of older people as a result of increasing life expectancy, it has recently been predicted that within 2050 more than half of the osteoporotic fractures around the world will occur in Asia (Rahnavard et al., 2009). The mortality rate of hip fractures among elderly Iranians is almost 20%, and 50% of those who remain alive have perennial disability for their remaining years of life (Larijani et al., 2004).

Common risk factors for osteoporosis are genetics, race, advancing age, smoking, alcohol consumption, lack of exercise, bad nutrition habits, calcium balance disorders, and many other unknown factors (Larijani et al., 2007; Maalouf et al., 2007).

The best way to prevent the complications of osteoporosis is encouraging people to modify their nutrition habits, to increment their intake of calcium and vitamin D, and to increase their physical activity (Costa et al., 2013).

Since everyone’s peak bone mass is influenced by several factors such as genetics, race, nutrition (Porhashem et al., 2012), and geographic region, the prevalence of osteoporosis is changing around the world. Considering the geographical variety of different parts of Iran, which is located north of the equator, and climatic conditions due to less exposure to sunlight and areas with diverse climate such as hot and dry, temperate, cold, rain, desert, is one of important factors that effects on the different prevalence of osteoporosis in different regions of Iran (Bagheri et al., 2011), and from the cultural aspect, Iranian women spend less time outdoors in comparison with men, which makes them more susceptible to the vitamin D deficiency and clothing habits in Iranian women is the other risk factor for osteoporosis (Tabrizi & Pakdel, 2014). This study aimed to estimate the prevalence and risk factors of osteoporosis for women who referred to bone density testing to the Urmia Imam Khomeini Academic Hospital, in the north-west of Iran.

## 2. Methods

### 2.1 Study Design, Participants and Sampling

This cross-sectional study was performed on 360 women, selected by a simple sampling method, who referred for bone density testing to the Urmia Imam Khomeini Academic Hospital in the north-west of Iran from February 2013 to June 2014. The data gathering process was carried out by the researchers by means of an interview after BMD measurement. Inclusion criteria were female gender, age above 15 years, and non-pregnant. The participants in the study were divided into two subgroups: women 45 years old or younger and women over 45 years. We calculated the sample size for the study using the findings of a study conducted by Khani Jeihooni et al. (2013) and the following formula,


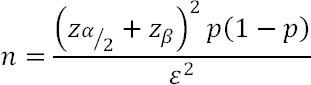



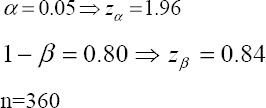


**Figure 1:**
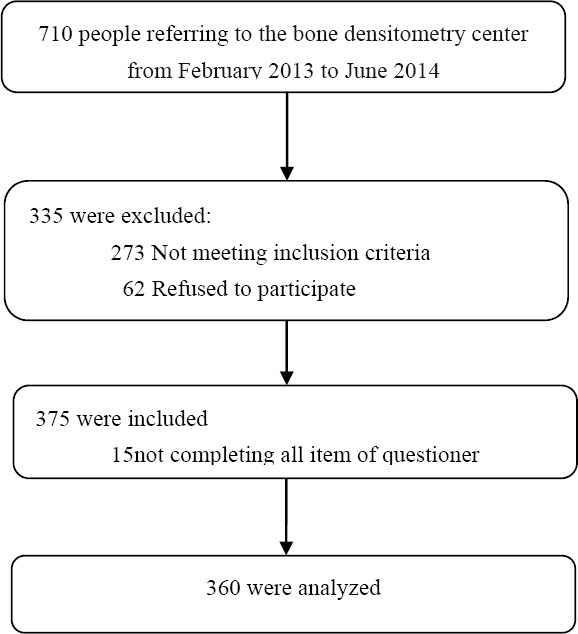
Flow chart diagram of the study

### 2.2 Ethics

The study commenced after the ratification from the Ethical Review Committee of the Shahid Beheshti University of Medical Sciences, in Tehran was obtained, [Code Number: 13447]. The aim of the study was explained to the participants, and a pre-test informed consent was obtained from all of them.

### 2.3 Research Tools

The data were collected using a questionnaire structured in two parts. Part one measured the baseline demographics characteristics (age, occupation, education, living place, income, weight, height, body mass index (BMI), marital status, gravidity, parity, age of menarche, duration of lactation, age of menopause, history of oophorectomy or hysterectomy before 45 years of age, past fracture history, family history of fracture and osteoporosis, hyperparathyroidism, renal diseases, diabetes mellitus, rheumatoid arthritis, chronic liver disease, chronic mal-absorption syndrome and thyroid disorder, medication intake of warfarin, calcium, hormone replacement therapy, corticosteroids, or thyroid hormones). Part two consisted of a lifestyle questionnaire: it measured alcohol consumption, smoking status, consumption of green tea, sun exposure (categorized as ‘none’, less than 15 minutes a day, or more than 15 minutes a day), and nutrition status. The Persian version of the standard Health Promoting Lifestyle-II (HPLP II) was used to collect the data on the nutrition dimensions of lifestyle, and the questions were evaluated by a Likert scale ranging from never (one) to routinely (four). The reliability and validity of this scale has been previously approved by several studies (Erfan Aubi et al., 2012; Mohammadi Zeidi et al., 2012). The BMD was measured at the femoral neck and the lumbar spine L1-L4 using a Hologic machine and the DEXA (dual-energy X-ray absorptiometry) technique. The DEXA scan report was based on the WHO classification ranges of T-score to classify the participants into three groups: normal (T-score ≥ −1 SD), osteopenia (−2.5SD < T-score < −1SD), and osteoporosis (T-score ≤ −2.5 SD) (Woolf & Pfleger, 2003).

### 2.4 Statistical Analysis

The data were analyzed using the SPSS software version 17.0. The results of the description of the study population were expressed in terms of frequency (percent) or as a mean (SD). Chi-squared tests were used for the nominal variables, and student’s t-test and one-way ANOVA were used for the quantitative variables. To determine the variables independently associated with osteoporosis, ordinal regressions were conducted; backward elimination was used to remove those variables found to be not significantly predictive of outcome. In all tests, a p value lower than 0.05 was considered significant.

## 3. Results

Three hundred and sixty women aged 15 years and older (mean ± SD, 53.35 ± 12.27) participated in this study; 23.3% of them were 15-45 years old and 76.7% were over 45 years. Osteoporosis was diagnosed in the 14.3% of the participants aged between 15 to 45 years and in the 50.7% of the participants over 45 years. The total prevalence of osteoporosis was 42.2%. The majority of the participating women were homemakers (74.6%), 96.7% were married, 28.6% were illiterate, and 69.2% had sufficient income. Among the participants, 67.1% were menopausal, 2.6% were reported to be current or past smokers, and 19.5% were reported to have taken corticosteroids within the last six months. 7.3% were reported to have taken thyroxin, 5.1% were reported to have taken estrogen, and 14.4% were reported to have taken calcium. None of the subjects had a history of alcohol intake. 9.4% of the participants resided in rural areas and 90.6% in urban areas. The prevalence of osteoporosis in urban areas was found to be higher than in rural areas, but not significantly (89.5% versus 10.5%; p=0.8). Housewives were more likely to develop osteoporosis (78.3%) compared with working (5.3%) and retired (16.4%) women (p<0. 05). The prevalence of osteoporosis among illiterate women was found to be significantly higher (43.5%) than among others (p<0.001).

The mean age, weight, height, gravidity, parity, duration of lactation (in months), and consumption of green tea (in cups) and nutrition dimension of lifestyle were statistically significant (P<0.05) in the osteoporosis and normal groups (Table 1).

**Table 1:** Baseline demographic characteristics of the study participants

Variables	Normal mean (SD)	Osteoporosis mean (SD)	p-value
Age (years)	47.27(12.7)	58.9(10.6)	0.001^b^
Weight (kg)	74.21(12.17)	69.29(3.38)	0.001^b^
Height(cm)	156.75(4.81)	153.37(6.64)	0.001^b^
BMI	30.13(4.83)	29.45(5.41)	0. 6^b^
No. of gravidity	3.42(1.85)	4.92(2.67)	0.001^a^
No. of parity	2.67(1.5)	4.26(2.04)	0.001^a^
Duration of lactation (month)	43.13(41.98)	70.04(60.89)	0.001^a^
Consumption of green tea (cup)	0.37(0.82)	0.17(0.6)	0.02^a^
Nutrition score	24.84(3.26)	23.53(3.7)	0.03^a^

a *Note.* P-value for Kruskal Wallis Test

b P-value for ANOVA test, BMI: body mass index.

Education level (p<0.001), history of bone fracture (p<0.001) and, history of diseases (rheumatoid arthritis, diabetes, and high blood pressure) (p<0.001), showed statistically significant associations with the BMD (Table 2).

**Table 2:** Qualitative variables surveyed

variables	Normal N (%)	Osteoporosis N (%)	P-value
Education			
Illiterate	7(11.1)	66(43.5)	<0.001^a^
Less than high school	25(39.7)	37(24.3)	
Diploma	15(23.8)	33(21.7)	
University educated	16(25.4)	16(10.5)	
Income			
Sufficient	50(79.4)	98(64.5)	0.08^a^
Less than sufficient	13(20.6)	54(35.5)	
Occupation			
Working	11(17.5)	8(5.3)	0.012^a^
Homemaker	48(76.2)	119(78.3)	
Retired	4(6.3)	25(16.4)	
Place of living			
Urban	57(90.5)	136(89.5)	0.8^a^
Rural	6(9.5)	16(10.5)	
History of diagnosis of osteoporosisin any parent or grandparent	13(20.6)	33(21.7)	0.61^a^
History of hip fracture in any parent or grandparent			
History of any fracture in yourself	3(4.8)	10(6.6)	0.79^a^
Past or current smoking	10(15.9)	44(28.9)	
History of menopause age before 45 years	1(1.6)	6(3.9)	0.001^a^
History of menarche age above 15 years	9(25.9)	47(34.3)	0.63^b^
History of oophorectomy before 45 years of age	8(12.7)	20(13.2)	0.16^a^
History of hysterectomy before 45 years of age	2(4.9)	7(5)	0.73^a^
History of Hypertension	2(4.9)	7(5)	0.37^b^
History of Rheumatoid arthritis	13(13.7)	49(51.6)	0.65^b^
History of Diabetes	12(15.5)	36(46.8)	<0.001^a^
Sun exposure	5(7.9)	<0.001^a^	
Less than 15 minutes a day		15(9.8)	<0.001^a^
More than 15 minutes a day	18(28.6)	46(30.3)	0.56^a^
Non exposure	25(39.7)	52(34.2)	
	20(31.7)	54(35.5)	

a *Note.* P-value for χ^2^ test

b P-value for Fisher’s exact test.

Among all the variables, the regression model showed that age (OR=1.08; for every 10-years increase in age, the risk of osteoporosis increased 2.18 fold), history of bone fracture (OR=2.75; having a history of bone fracture increased the risk of developing osteoporosis 2.75 fold), and gravidity (OR=1.14; each pregnancy increased the risk of osteoporosis by14%) were identified as significant risk factors for osteoporosis, and body mass index (OR=0.94; for each unit increase in body mass index, the risk of osteoporosis was reduced by 0.05) was identified as a protector against osteoporosis (Table 3).

**Table 3:** Predictors of osteoporosis obtained by ordinal regression analysis (n=360)

Variables	Beta	Wald	Exp(B)	p-value
Age	0.78	30.176	1.08	<0.001
Body mass index	-0.58	5.288	0.944	0.021
No. of pregnancy	0.135	4.654	1.145	0.031
History of fracture	1.014	10.485	2.75	0.001

## 4. Discussion

In the current study, the total prevalence of osteoporosis was found to be 42.2%, and osteoporosis was found in 14.3% of the participants aged 15- 45 years old and 50.7% of the participants over 45 years of age. A recent study in women (aged 45 years and older) in India found a prevalence of low BMD in 53% of the population (Aggarwal et al., 2011). Another study related to Saudi Arabia showed that 18% of the women(without hormonal disorders and renal diseases) in the 40-75age group had osteoporosis (Anitha & Ibrahim, 2014), and a study performed in Pakistan among women 25 years old or over found instead that 49.3% of post-menopausal women and 17.8% of premenopausal women were osteoporotic (Ejaz, Mahmood, Qureshi, & Ali, 2012). In Iran, as a result of the demographic transition and the increasing age of the population, osteoporosis has become an important public health issue (Meybodi et al., 2008). Women of the Asian race have lower bone mass than white women (Barrett-Connor et al., 2005). Vitamin D is also an essential factor for this silent diseases prevention; Unfortunately, the prevalence of vitamin D deficiency in Iran is high (Heshmat et al., 2008). Discrepancies in reports from different countries are significantly affected by differences in nutrition, genetics, lifestyle, and geographic region as well as used the diagnostic techniques.

Among the variables investigated in the our study, age, history of bone fracture, and gravidity were identified as significant risk factors for osteoporosis, while the body mass index was identified found to act as a protector against it. Results also showed that with every 10-years increase in age, the risk of osteoporosis was increased 2.18 fold, a history of bone fracture increased the risk 2.75 fold, and for every unit increase in body mass index, the risk of osteoporosis was reduced by 0. 05.

In the following, the investigated parameters will be separately discussed. The results of our study will be compared with the literature.

A study by Pinheiro et al. (2010) showed that, with the advancement of age and having a history of prior bone fracture, the risk of having low BMD was increased and a higher BMI protects against low BMD (Pinheiro et al., 2010).

Results of the current study demonstrated that gravidity, parity, and duration of lactation were significantly higher in the osteoporosis group than in the normal group. An association between parity and bone loss was reported also by Allali et al. (2007), while another study showed that parity had no association with osteoporosis (Parazzini et al., 1996). One results showed that, during pregnancy, there is a change in levels of insulin-like growth factor 1 (IGF-I), which has an important role in the bone turnover causing trabecular bone loss in pregnant women (Naylor et al., 2000). The duration of lactation in some studies has been shown to have no association with osteoporosis (Afzal et al., 2013; Aksakal et al., 2008), but in other studies of postmenopausal women, the duration of lactation was instead found to have a negative correlation with the BMDs of the femoral neck and the lumbar spine (Hosseinpanah, Sorouri, Rambod, & Azizi, 2011). It was estimated that almost the 3-7% of a woman’s bone density is temporarily decreased during the lactation period; fortunately, it is regained after weaning (Kalkwarf & Specker, 2002).

In the current study, no statistically significant difference was seen between osteoporosis and history of menarche age above 15, menopause age before 45 years, or history of oophorectomy or hysterectomy before 45 years. Both Parazzini et al. (1996) and Jamshidian-Tehrani et al. (2004) showed no relationship between the menarche age and BMD (Jamshidian-Tehrani et al., 2004; Parazzini et al., 1996), while in another study, early menarche in women 21 to 74 age group was significantly associated with high BMD (Ito et al., 1995). These contradictions may be related to the fact that some women may not remember or remember inaccurately the exact date of their menarche. In the postmenopausal women, the main factor for predicting the osteoporosis is age (Koh, Park, & Kim, 2012), and normal body weight has been shown to have a key role in bone health in younger postmenopausal women (Gourlay et al., 2014).

The peak bone mass is a determining factor for bone health and future chance of osteoporosis; one standard deviation increase in peak bone mass can reduce the fracture risk by one half (Bonjour et al., 2009). One study showed that the risk of bone loss in women is elevated 15 years after beginning menopause (Sharmai, 2008). It is passible that participants in this study had a lower number of years after menopause. In a study conducted by Fletcher et al. (2013), hysterectomy in black Jamaican postmenopausal women was not a significant risk factor for osteoporosis (Fletcher et al., 2013). Furthermore, Sharmai et al. (2008) showed that oophorectomy was not associated with osteoporosis (Sharmai, 2008). In surgically-induced menopause, osteoclast cell activity is stimulated as a result of increased secretion of cytokines (Pacifici et al., 1991). It is also possible that the dysregulation of levels of nutrients such as magnesium, calcium, and phosphorus after hysterectomy increases the risk of osteoporosis (Sreekantha et al., 2011). In the current study the number of women with a history of oophorectomy or hysterectomy before the age of 45 was very low.

In contrast with the results of studies conducted by Hollenbach et al. (1993) and Kidambi et al. (2005) (Hollenbach, Barrett-Connor, Edelstein, & Holbrook, 1993; Kidambi, Partington, & Binkley, 2005), the results of the current study indicated that past or current smoking habits were not significantly associated with osteoporosis. This may be because the number of smoking participants in this study was low. One study demonstrated that smoking decreased the level of estrogen hormone and led to bone loss in women (Vogt, 2000).

In the current study, a high prevalence of osteoporosis in some medical conditions such as rheumatoid arthritis, diabetes, and high blood pressure was reported by subjects and was significantly associated with the bone mineral density. Whether the risk of osteoporosis is increased in diabetic patients is a controversial issue (Leidig-Bruckner et al., 2014). One study showed that patients with diabetes mellitus type 1 had a reduced BMD for lumbar spine and proximal hip compared with controls (Botushanov et al., 2014). The use of corticosteroids, an anti-inflammatory drug which reduces the production of (IL-1 and IL-6), led to osteoporosis in patients with rheumatoid arthritis (RA) (Geraci, 2012). A recent study showed that in hypertension disorder, some factors such as low calcium intake, vitamin D and vitamin K deficiencies, and high consumption of sodium can lead to osteoporosis, and different anti-hypertensive drugs can have either a negative or positive influence on bone mineral density (Ilić, Obradović, & Vujasinović-Stupar, 2013).

In our study, the consumption of green tea was significantly higher in the normal group than in the osteoporosis group. There is evidence that the antioxidant and anti-inflammatory properties of green tea have a protective effect against osteoporosis (Shen et al., 2011; Shen et al., 2009).

In this study, the mean score of nutrition dimension of lifestyle was higher in the normal group than in the osteoporosis group. Mahdaviroshan et al. (2014) found that osteoporotic patients do not have good nutrition habits in their life (Mahdaviroshan & Ebrahimimameghani, 2014). The nutrition habits play a key role in the prevention and treatment of osteoporosis (Rafraf & Bazyun, 2011), Improving the lifestyle of women, especially their nutrition dimension of lifestyle, would reduce the economic and health costs of osteoporosis.

In the current study, the sun exposure was not significantly associated with BMD. Maeda et al. (2007) showed that sunlight exposure can significantly increase 25OHD levels (Maeda et al, 2007), while in another study, the sunlight exposure did not affect serum vitamin D (Ataie-Jafari et al., 2008). A possible explanation for these antithetic results might be offered by the different clothing habits among women of related to this region, The clothing habits are indeed believed to be an important factor in vitamin D deficiency. It is also possible that women of the Asian race may have a lower capacity to synthesize vitamin D from the sunlight (Ataie-Jafari et al., 2008).

## 5. Conclusion

The current study found the prevalence of osteoporosis to be high and the advancement of age, lifestyle, and reproductive factors, especially gravidity and duration of lactation, to have detrimental effects on the women’s bone mineral density. Appropriate educational programs and interventions may ultimately reduce the risk of developing osteoporosis for women.

**Limitation**

Due to the high cost of studying the general population, the presented study focused on women who were admitted to the Urmia Imam Khomeini Hospital Bone Densitometry Center. A generalizing of the results to the entire population should be performed with caution.
